# Rheumatoid Arthritis Affecting the Upper Cervical Spine: Biomechanical Assessment of the Stabilizing Ligaments

**DOI:** 10.1155/2017/6131703

**Published:** 2017-10-18

**Authors:** Carolin Meyer, Jan Bredow, Elisa Heising, Peer Eysel, Lars Peter Müller, Gregor Stein

**Affiliations:** ^1^Department of Orthopedics and Trauma Surgery, University Hospital of Cologne, Kerpener Str. 62, 50937 Cologne, Germany; ^2^Center for Spinal Surgery, Schön Klinik Düsseldorf, Am Heerdter Krankenhaus 2, 40549 Düsseldorf, Germany; ^3^Medical Department, Luzerner Kantonsspital, Spitalstrasse 16, 6000 Luzern, Switzerland; ^4^Department of Orthopedics and Trauma Surgery, HELIOS Klinikum Siegburg, Ringstraße 49, 53721 Siegburg, Germany

## Abstract

Diameters of anterior and posterior atlantodental intervals (AADI and PADI) are diagnostically conclusive regarding ongoing neurological disorders in rheumatoid arthritis. MRI and X-ray are mostly used for patients' follow-up. This investigation aimed at analyzing these intervals during motion of cervical spine, when transverse and alar ligaments are damaged. AADI and PADI of 10 native, human cervical spines were measured using lateral fluoroscopy, while the spines were assessed in neutral position first, in maximal inclination second, and in maximal extension at last. First, specimens were evaluated under intact conditions, followed by analysis after transverse and alar ligaments were destroyed. Damage of the transverse ligament leads to an increase of the AADI's diameter about 0.65 mm in flexion and damage of alar ligaments results in significant enhancement of 3.59 mm at mean. In extension, the AADI rises 0.60 mm after the transverse ligament was cut and 0.90 mm when the alar ligaments are damaged. After all ligaments are destroyed, AADI assessed in extension closely resembles AADI at neutral position. Ligamentous damage showed an average significant decrease of the PADI of 1.37 mm in the first step and of 3.57 mm in the second step in flexion, while it is reduced about 1.61 mm and 0.41 mm in the extended and similarly in the neutrally positioned spine. Alar and transverse ligaments are both of obvious importance in order to prevent AAS and movement-related spinal cord compression. Functional imaging is necessary at follow-up in order to identify patients having an advanced risk of neurological disorders.

## 1. Introduction

Rheumatoid arthritis (RA) is a common chronic, systemic autoimmune disease, affecting an estimated 1-2% of the global population, being three times more frequent in women [1]. Leading to synovitis, RA comes along with hypertrophy of the synovial tissue as pannus formation. Distinctively, these inflammatory, symmetric processes occur at peripheral joints, resulting in erosion of the articular cartilage and subchondral bone [2, 3].

The cervical spine also represents a predilection site of RA, being the most common inflammatory disorder affecting the spine [2]. Ordinarily, upper cervical spine involvement starts in the early course of disease. Affection of the lower cervical spine, however, is less common and arises in the later course of disease, manifesting as multilevel dislocation [4]. Regarding progression of RA-related spinal disorders, a close correlation to the severity as to the extent of disease's activity at peripheral joints was proven [5, 6]. Overall, the rapidity of progression is highly variable. Previous investigations pointed out that men have major risk of advanced cervical spine involvement [2, 7, 8].

**Figure 1:**
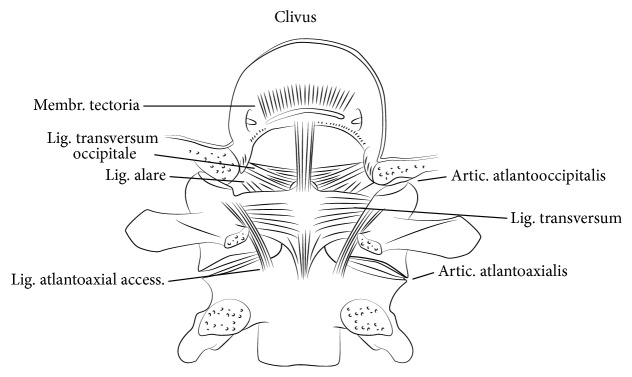
The atlantoaxial complex in the dorsal view.

**Figure 2:**
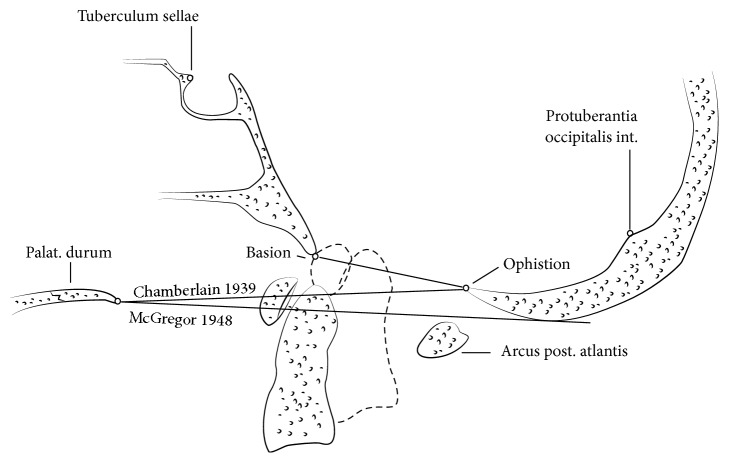
Radiological ledger lines for assessment of RA-related unstable disorders of the upper cervical spine.

Due to erosions of the transverse, the apical, and the alar ligaments, anterior atlantoaxial subluxation (AAS) develops. Damage to the transverse ligament alone leads to approximately 3-4 mm of subluxation. A greater anterior atlantodental interval (AADI), however, requires damage to the alar and apical ligaments, potentially combined with changes of the bony shape of the dens axis (Figure 1) [9]. If the odontoid process is involved as the bone erodes, posterior AAS possibly appears. Initially, these changes, still representing a stable situation, may require stretching of surrounding neural and vascular structures during extension of the spine. At least fracturing or disappearance of the odontoid process combined with damage of the atlantoaxial joints results in posterior atlantoaxial dislocation, causing ventral compression of the spinal cord by C1 [10]. If, additively, lateral mass joints and lateral masses of occiput, atlas, and dens axis are degraded and progressively damaged, at least atlantoaxial impaction occurs due to a complete collapse of the lateral masses determining craniocervical instability and resulting in occasional ankylosis coming along with a spontaneous stabilization of C1 and C2 [11]. Thus, superior migration of the odontoid process towards the foramen magnum proceeds, while even sometimes the border of the foramen is crossed (Figures 1 and 2). Consequently, cranial settling, basilar invagination, and compression of the spinal cord or even the brain steam is inducted, resulting in ischaemic or compressive related neurological impairment or even death [8, 12–14].

For diagnosis, lateral X-rays are reliable for the investigation of the relevant parameters of the craniocervical junction. MRI provides further information especially on ligamentous structures.

In lateral X-rays of the inclined cervical spine, anterior AAS is diagnosed, if the AADI, measured as the interosseous distance between the posterior aspect of the arch of the atlas and the anterior aspect of the odontoid process, exceeds 2.5 mm [15]. If the posterior aspect of the anterior arch of the atlas appears posteriorly to the physiological line of the anterior border of the axis, posterior AAS is diagnosed in lateral X-rays of the extended spine [10]. Previous investigations declared that the posterior atlantodental interval (PADI), depicting the distance between the posterior surface of the odontoid and the anterior margin of the posterior ring of the atlas, which regularly measures 19–27 mm, is diagnostically more conclusive regarding the risk of spinal cord compression and resulting neurological disorders [4, 16, 17].

In this investigation, analyses were performed in order to expose how far AADI and PADI alter at physiologic, age-related movement, if alar and transverse ligaments are damaged or get insufficient. The goal of this study was to determine stabilizing values of the ligaments of the atlantoaxial complex firstly in order to derive their contribution preventing neurological impairment secondly.

## 2. Materials and Methods

Ten native, frozen, human specimens (C0 to Th1) from informed and consented body donors were available for simulation of ligamentous disorders, analogical to RA-related ligamentous degeneration, and assessment of alterations at the spinal canal as a consequence. Average age of the body donors was 84.6 years; there were eight female body donors.

First, specimens were assessed radiologically to exclude present, advanced, and radiologically clearly apparent degenerative disorders or rheumatoid changes at level C1/C2; further examination of the cartilage layers was not performed [14, 18, 19].

An experienced spine surgeon dissected specimens as the surrounding soft tissue of the cervical spine was removed, while the paravertebral ligaments were kept protected. Thereby, either anterior or posterior longitudinal ligaments were left intact as well as the joint capsules C0/C1 and C1/2 and the atlantoaxial and atlantooccipital ligaments. Furthermore, neural structures were removed from the spinal canal in order to get access for further preparation during testing.

The first thoracic vertebral body was fixed in place, while the occiput was placed into a gadgetry allowing movement of the spine in sagittal plane only.

Initially, the force, which is necessary to move the cervical spine to age-related, regular range of motion, meaning 50° of extension and 40° of flexion, was determined [20]. The force needed to achieve movement of the cervical spine in this range was assessed to account for 30 N.

Specimens were moved to flexion and extension three times, respectively, in order to reduce viscoelastic effects as potential bias.

**Figure 3:**
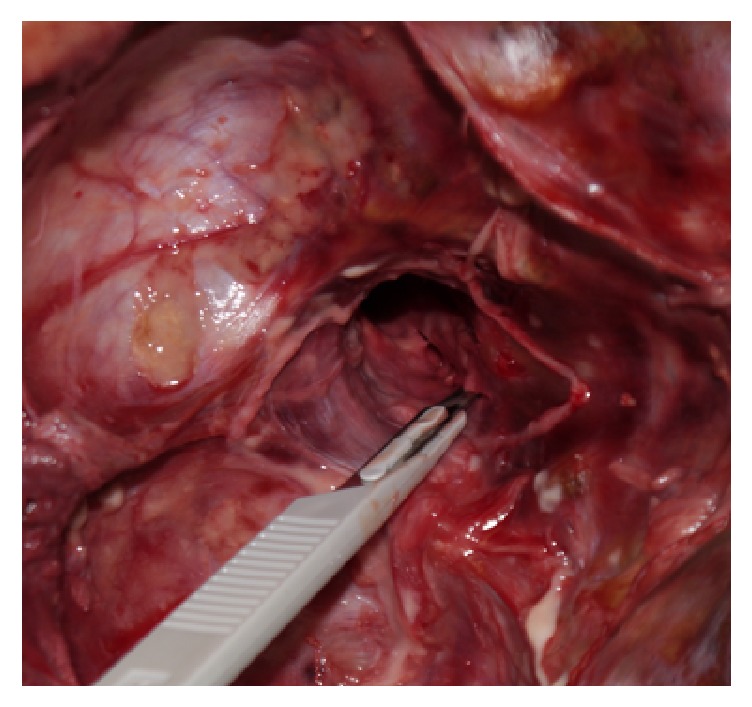
Transspinal approach used to cut the alar ligaments and the transverse ligament.

Initially, testing was performed using specimens with intact ligaments as vertebrae. Afterwards, the transverse ligament was cut firstly and the alar ligaments were destroyed secondly using a transspinal approach (Figure 3).

Thereafter, testing was performed in the same way at each specimen. The previously assessed force of 30 N was applied to the occiput in flexion direction firstly and in extension direction secondly crossing neutral position in the presence of stringent sagittal fluoroscopy. Radiologically produced images at the points of maximal flexion and extension at neutral position were analyzed. AADI and PADI were assessed using a horizontal center line through the atlas. A standardized radiologic marker was introduced in order to minimize inaccuracy of measurement caused by fluoroscopy related inexactness.

Measurements were carried out by three investigators independently, as images were analyzed by a medical student, a resident, and a chief resident. Ascertained results of the three investigators were averaged. Thereafter, the results were evaluated using spreadsheet software (Microsoft Excel, MAC 2011), validating normal distribution by Kolmogorov-Smirnov and performing Student's* t*-test and ANOVA (VassarStats); *p* values ≤ 0.05 were considered to be statistically significant.

## 3. Results

### 3.1. Anterior Atlantodental Interval

The AADI in intact specimen measured 1.62 mm ± 0.62 on average. At the point of maximal flexion, AADI extended 1.88 mm ± 0.85 at mean. In extension, average width amounted to 1.63 mm ± 0.61.

Once the transverse ligament was cut, AADI in neutral position of the cervical spine altered to 2.19 mm ± 0.58. Applying force of 30 N to achieve flexion, the extent of the AADI changed to 2.53 mm ± 1.11 at mean. In extension position, AADI was reduced to 2.23 mm ± 0.57 on average.

**Figure 4:**
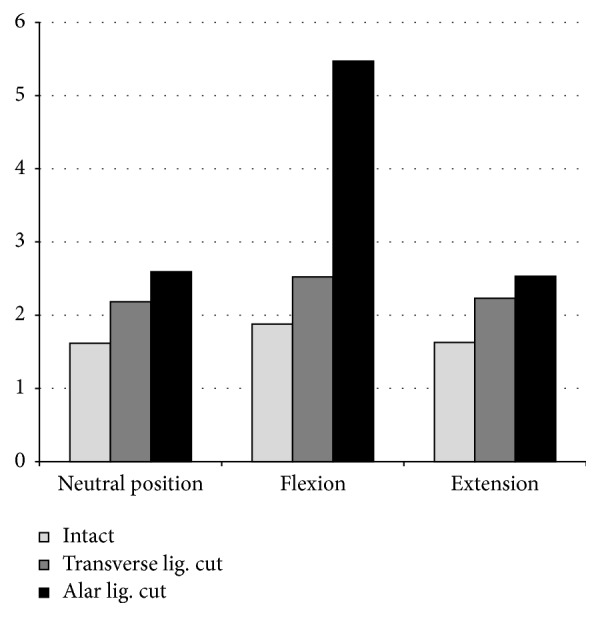
AADI measured in mm.

After the alar ligaments were destroyed, the average width of the AADI amounted to 2.59 mm ± 0.84 at neutral position and to 5.47 mm ± 1.23 (*p* < 0.05) in flexion and comes up to 2.53 mm ± 0.58 in extension on average (Figure 4 and Table 1).

### 3.2. Posterior Atlantodental Interval

The extent of the PADI in intact specimen ranged from 17.57 mm minimally to 20.86 mm maximally in neutral position of the cervical spine, averaging 18.85 mm ± 1.25. At maximal achieved flexion, the average width accounted to 18.49 mm ± 2.88. Images of maximal extension of the cervical spines of intact specimen showed an average PADI of 18.91 mm ± 2.65.

Once the transverse ligament was cut, the PADI measured 18.60 mm (SD: 2.90) on average, whereas diameter altered significantly to 16.20 mm (SD: 3.10) in flexion and to 17.30 mm ± 3.10 (*p* = 0.05) in extension.

**Figure 5:**
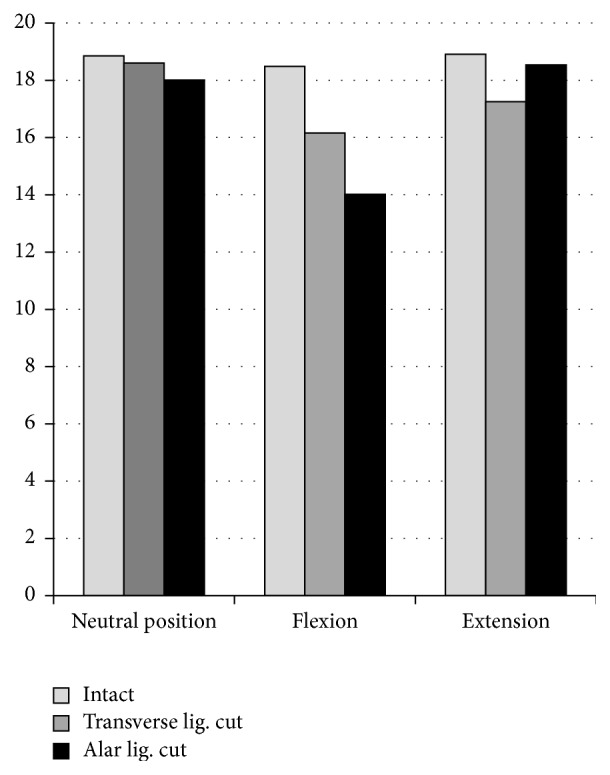
PADI measured in mm.

After the alar ligaments were destroyed as well, diameter of the PADI increased to a mean of 18.00 mm ± 2.70 in neutral position and altered significantly (*p* = 0.04) in contrast to values assessed in specimen, if only the transverse ligament was cut. Moreover, PADI increased to 14.00 mm ± 2.00 on average in flexion, differing significantly from values measured in intact specimen (*p* = 0.03). Minimal PADI measured at the inclined spine after transverse and alar ligaments were destroyed accounted for 11.60 mm (Figure 5). It altered to an average of 18.50 mm ± 3.10 in the extended spine (Table 1).

## 4. Discussion

Based on our present findings, we conclude that alar and transverse ligaments are of obvious importance, retaining the stability of the occipital-atlantoaxial complex. Damage of these ligamentous structures leads to radiological changes, possibly related to neurological impairment.

The extents of the AADI assessed in intact specimens correspond to physiological values recorded in the literature [4, 16, 17]. If only the transverse ligament is destroyed, extent of the AADI alters to more than 2.5 mm in some cases in neutral position and beginning AAS would be diagnosed. If cervical spine is flexed to the age-related, regular range of motion of 40°, the AADI increases (2.53 mm on average) [20]. Furthermore, in some cases, the AADI does not decrease to a physiological width at 50° extension position. If patients, whose transverse and alar ligaments are eroded, incline their cervical spine, the AADI broadens excessively (5.47 mm on average). Supporting the diagnosis of anterior AAS in every case, an extent of greater than 5 mm represents an obvious and clinically relevant instability of C1 and C2. Our findings demonstrate that ligamentous damage solitarily results in a pattern, affecting 24 to 65% of patients suffering from RA, resulting in myelopathy in 5% [9].

The PADI, regularly measuring 19 to 27 mm, is slightly reduced in recent intact specimens in neutral position (18.85 mm on average: 17.57 mm to 20.86 mm) [4, 16, 17], which is most likely induced by the advanced age of the used cervical spines. Once the transverse ligament was damaged, diameter of the PADI was reduced significantly to 13.00 mm in flexion and to 13.70 mm in extension minimally. Eroding the alar ligaments as well, the PADI decreases excessively again inclining the cervical spine (14.00 mm at mean), while the smallest PADI assessed extended to 11.60 mm.

The present results highlight that AADI is increased and PADI is decreased even at neutral position after the ligaments were cut. These data suggest that the position of the anterior aspect of the odontoid process at the posterior border of the anterior arch of the atlas is mainly stabilized by both ligaments. During extension, the extent of AADI approximately measures the same as in neutral position, because the anterior arch of the atlas moves back to the side of the anterior aspect of the axis; however, the ligaments are destroyed.

Several authors declared that AAS greater than 9 to 12 mm and a minimized PADI of 14 mm or less positively correlate with neurological disorders, even if surgical stabilization and decompression are performed at this point [4, 18, 21]. Moreover, authors found out that the preoperative diameter of the posterior atlantodental interval is the most important predictor of the potential for neurological recovery postoperatively. AAS greater than 9 mm with VT and a posterior atlantodental interval, measuring less than 14 mm, correlate with continuance of neurologic deficits positively [4, 16, 17].

Our results show significant enlargement of the AADI due to damage of the alar and transverse ligaments, although it was demonstrated that the capsules and the articular surfaces of the atlantoaxial joints as the anterior and posterior longitudinal ligaments ensure enough stability to prevent complete atlantoaxial dislocation [22].

Results of the assessment of the PADI, however, suggest repeated spinal cord compression during movement of the cervical spine in some cases, if the transverse ligament is expanded or destroyed. If the alar ligaments are damaged additively, spinal cord compression occurs in 50% of the cases during flexion procedures of the cervical spine.

Clinical observations showed that the above-mentioned, radiological abnormalities can remain asymptomatic for years in many patients. Most commonly, patients present unspecific symptoms like occipital headache or migraine as high neck, mastoid, ear, or facial pain [2, 9, 23, 24]. Asymptomatic and misunderstood patients, however, do run the risk of myelopathy or even sudden death, though conservative treatment with disease-modifying antirheumatic drugs and biologicals is achieved preventing progress of spinal changes [18, 25, 26]. Our findings show that even in some clinically asymptomatic patients repeated spinal cord compression at levels C1 and C2 has to be expected, possibly causing myelopathy [2, 9, 23, 24].

Overall, the incidence of severe radiologic changes of the cervical spine and neurologic disorders is reduced considerably by modern treatment strategies, ranging from paresthesias to weakness and from missing endurance and gait disturbance to loss of dexterity. Vertebrobasilar insufficiency gets clinical evidence by tinnitus and vertigo and loss of equilibrium and visual disorders or diplopia and dysphagia [2, 8, 9]. Today those disorders nearly only affect patients with chronic, severe, and erosive RA [25, 27]. However, once the patient becomes myelopathic, the rate of long-term mortality increases and the chance of neurologic recovery decreases [28].

Due to missing symptoms in many patients presenting cervical involvement as signs of instability radiologically as the perioperative risk on one hand and the advanced neurological deficits correlating to increased morbidity and mortality on the other hand, controversial discussion about the appropriate timing for surgical treatment persists [29]. Many authors, however, recommend early and aggressive surgical intervention before the onset of neurological symptoms in patients with radiological signs of instability appears to occur, if only the ligaments get insufficient but the bone, the joints, and the masses remain intact, as shown in the present investigation [18, 28, 30–32].

In the early course, disorders of the atlantoaxial complex are not fixed yet. Therefore, a surgical intervention could restore the physiological and anatomical alignment completely. Moreover, surgical treatment at an early stage of rheumatic disease appears to be easier to perform and it is less extensive, whereas surgical interventions will be more complicated due to advanced radiological disorders or even added neurological abnormalities in the further course [18].

The time of onset of radiological abnormalities or specific symptoms for involvement of the cervical spine is variable. Nevertheless, a correlation between the aggressiveness of RA at peripheral joints and the intensity of cervical spine involvement has been revealed [23, 33]. Some authors advise computer tomographic check-ups regularly to detect pathologic changes at the atlantooccipital and atlantoaxial joints [34], though MRI appears to be the gold-standard method in order to detect inflammatory processes at the early course and spinal cord alterations during the further course [13, 35].

Several limitations have to be mentioned. Firstly, X-rays are summation images possibly leading to inaccurate identification of the cortical borders of atlas and axis. Nevertheless, it is mostly used in daily routine for follow-up [16]. Investigators tried to minimalize inaccuracy performing absolutely lateral fluoroscopy. Secondly, cervical spines were analyzed at an in vitro setup, whereby muscles did not work as stabilizers anymore. Thirdly, joint capsules, cartilage, and bone also stabilizing the occipital-atlantoaxial complex were kept intact. These structures, however, are also eroded by RA in the natural course. At least it can be assumed that there were degenerative changes of the cartilage layers due to the aged donors biasing our results.

## 5. Conclusions

In a synopsis of the literature and our findings, we conclude that not only the alar but also the transverse ligaments are of obvious importance in preventing AAS and movement-related spinal cord compression. Our results support the available concept that functional imaging of the cervical spine is necessary during follow-up in order to assess existing instability, even if MRI of the cervical spine at neutral position does not show any affection of osseous and cartilaginous structures yet. Thereby, close neurologic investigations, about disorders of somatosensory evoked potentials, can be performed and the appropriate point for surgical intervention can be found in order to prevent myelopathy.

## Figures and Tables

**Table 1:** Diameter of AADI and PADI measured in mm at neutral position, flexion, and extension: measurements were carried out in intact specimens and after the transverse and the alar ligaments were damaged.

		Neutral position			Flexion			Extension		
		Average	Minimum	Maximum	Average	Minimum	Maximum	Average	Minimum	Maximum
PADI	Intact	18.85	17.57	20.86	18.49	15.46	22.89	18.91	15.81	23.02
	Transverse lig. cut	18.60	15.70	22.00	16.20	12.97	20.69	17.30	13.73	21.75
	Alar lig. cut	18.01	15.74	22.11	14.00	11.57	16.40	18.50	14.61	23.15
AADI	Intact	1.62	0.65	2.33	1.88	0.90	2.91	1.63	0.78	2.33
	Transverse lig. cut	2.19	1.80	3.18	2.53	1.55	3.92	2.23	1.54	2.84
	Alar lig. cut	2.59	1.74	3.81	5.47	3.75	6.99	2.53	2.02	3.38
